# Validation of the Arabic Version of the Multiple Sclerosis Impact Scale (MSIS-29): a Rasch Analysis Study

**DOI:** 10.1093/arclin/acae121

**Published:** 2024-12-27

**Authors:** Walid Al-Qerem, Dunia Basem, Sawsan Khdair, Anan Jarab, Judith Eberhardt

**Affiliations:** Department of Pharmacy, Faculty of Pharmacy, Al-Zaytoonah University of Jordan, Amman 11733, Jordan; Department of Pharmacy, Faculty of Pharmacy, Al-Zaytoonah University of Jordan, Amman 11733, Jordan; Department of Pharmacy, Faculty of Pharmacy, Al-Zaytoonah University of Jordan, Amman 11733, Jordan; College of Pharmacy, Al Ain University, Abu Dhabi 112612, United Arab Emirates; Department of Psychology, School of Social Sciences, Humanities and Law, Teesside University, Borough Road, Middlesbrough TS1 3BX, UK

**Keywords:** Quality of life, Multiple sclerosis, Assessment, Everyday functioning, Statistical methods, Test construction

## Abstract

**Background:**

Multiple sclerosis (MS) is a potentially disabling disease of the brain and spinal cord. This cross-sectional study aimed to validate the Arabic version of the Multiple Sclerosis Impact Scale-29 (MSIS-29) using Rasch analysis to assess quality of life in Jordanian MS patients.

**Method:**

Rasch analysis was conducted to evaluate the suitability of the model for the present study. Model fit was assessed by computing item/person separation reliability, infit and outfit mean square (MSQ) values, Cronbach's alpha, and the Akaike Information Criterion.

**Results:**

A total of 301 MS patients were enrolled in the study. Significant likelihood ratios for all three scales (MSIS-29-PHYSICAL, MSIS-29-PSYCHOLOGICAL, and MSIS-29-TOTAL) supported the use of a partial credit Rasch model. An issue with disordered thresholds was resolved by collapsing adjacent response categories. Item reliability scores for MSIS-29-PHYS and MSIS-29-PSYCH were 0.95 and 0.89, respectively, while person reliability scores were 0.92 and 0.84, respectively. Infit and outfit MSQ were within the acceptable range for all items on the MSIS-29-PSYCH scale. However, for the MSIS-29-PHYS scale, item MSIS-29_17 exceeded the acceptable range in both infit (1.93) and outfit (1.82) MSQs, and item MSIS-29_20 exceeded the acceptable range in infit (1.81). The Wright map also indicated that most items were considered relatively easy by the respondents, exhibiting various difficulty levels on the latent scale.

**Conclusion:**

The Arabic version of the MSIS-29 is a valid and reliable tool for evaluating quality of life in Jordanian MS patients.

## INTRODUCTION

Multiple sclerosis (MS) is a chronic autoimmune disease that causes demyelination and neurodegeneration in the central nervous system (CNS) (Goldenberg, 2012). Approximately 2.3 million people worldwide are affected by MS. The overall MS prevalence in the Middle East is 51.52 per 100,000 people (Heydarpour et al., 2015). MS is considered the second-most disabling chronic condition affecting the CNS (Rezapour et al., 2017). The exact cause of MS is unknown, but it is thought to result from a complex interplay of genetic and environmental factors. Despite the lack of a cure, disease-modifying therapies have shown some efficacy in preventing relapses and disability progression (Yamout & Alroughani, 2018). MS can lead to various symptoms, including fatigue, depression, urinary incontinence, vision impairment, balance issues, and language impairment. Fatigue is the most common and debilitating symptom (Induruwa et al., 2012).

Quality of Life (QoL) refers to the overall state of well-being of an individual or a population at a specific point in time, encompassing both positive and negative aspects of life (Teoli & Bhardwaj, 2023). There are numerous benefits of using QoL evaluations to measure outcomes in chronic illnesses. It is patient-centered, applicable across various medical specialties, and focuses on measuring gradual improvement rather than complete recovery. QoL takes into account a wide range of factors of daily life (Ysrraelit et al., 2017). Research has indicated that individuals with MS tend to have a lower QoL than both the general population and those with other chronic illnesses (Mitchell et al., 2004).

MS can impact QoL due to a variety of disease-related and individual characteristics, including age, employment, level of education, and social support (Hyarat et al., 2019). A crucial step in implementing measures to enhance patients' QoL is identifying risk and protective factors (Gil-González et al., 2020). The duration of the disease, progressive MS onset, and recent relapses within the past three months are all factors that negatively affect QoL (Rezapour et al., 2017). MS patients' QoL is evaluated using disease-specific questionnaires, which are designed to reflect the distinct challenges and effects of the condition on various aspects of everyday living, as well as with generic tools. The most used disease-specific tools are the Multiple Sclerosis Quality of Life-54 (Giordano et al., 2021), the Multiple Sclerosis Impact Scale (MSIS-29) (Hobart et al., 2001), the Functional Assessment of Multiple Sclerosis scale (Cella et al., 1996), and Guy's Neurological Disability Scale (Sharrack & Hughes, 1999). While these tools offer invaluable insights into patients' experiences, there is a gap in the availability of culturally and linguistically adapted versions for Arabic-speaking populations. The MSIS-29 was selected in the present study due to its concise format and focus on both physical and psychological impacts. The MSIS-29 was preferred due to its comprehensive coverage of the diverse effects of disease on the patient's QoL and its use of straightforward vocabulary (Hobart et al., 2001). It overcomes the limitations of other instruments that were not developed using the standard psychometric approach of reducing a large item pool generated from people with MS. Although a translated version of the MSIS-29 was recently published (Albishi et al., 2024), it has several significant limitations. The study enrolled only 60 patients, raising concerns about the validity, reliability, and generalizability of the results. Furthermore, it did not employ advanced statistical techniques, such as Rasch analysis, to assess the construct validity of the tool. Considering the diverse cultural factors and specific healthcare needs of Arabic-speaking MS patients, validating the MSIS-29 using advanced statistical methods on a larger sample could greatly improve the accuracy and relevance of QoL assessments in this population. Therefore, the present study aimed to validate the MSIS-29 to more effectively assess and address the distinct challenges faced by Arabic-speaking patients with MS.

## METHOD

### Design

A cross-sectional study was conducted at Al-Bashir Hospital in Amman from January to May 2024. Patients aged 18 years or older, diagnosed with MS for at least one year, were recruited for participation. After being contacted, participants completed the questionnaire via a Google Forms link sent through the messaging application WhatsApp. Ethical approval for this study was granted by the Research Ethics Committee of Al-Zaytoonah University of Jordan (approval number MOH/REC/2021/263), and all procedures adhered to the Declaration of Helsinki’s ethical guidelines.

### Instrument

The MSIS-29 is a 29-item self-report measure, comprising 20 items on a physical scale and nine items on a psychological scale. Each item assesses the impact of MS on day-to-day life over the past two weeks. The response scale for all items ranges from 1, indicating 'not at all,' to 5, indicating 'extremely'. The survey was translated into Modern Standard Arabic following the Brislin principle (Brislin, 1970) to ensure that the Arabic version maintained cultural relevance and preserved the original meaning throughout. Two independent translators produced separate Arabic versions of the questionnaire. The translators and researchers then compared the two versions, analyzing and resolving any discrepancies to create the initial draft of the Arabic version. This draft was subsequently back-translated into the original language by two different translators to ensure accuracy and fidelity. The researchers and translators reviewed the two back-translated versions, the original version, and the initial Arabic draft to develop the final Arabic version. This thorough process ensured the accuracy and cultural appropriateness of the final translation.

In addition to the original questionnaire, a questionnaire was developed to gather demographic information about the participants, including age, gender, income, marital status, and level of education. All questions in the Google form were mandatory; therefore, participants could not submit the questionnaires without completing all the questions, which eliminated any missing values.

### Sample and recruitment

A total of 800 phone numbers for MS patients were obtained from Al-Bashir Hospital. However, ~80 patients did not respond, and 400 patients had changed their phone numbers. Ultimately, 321 patients were enrolled in this study. Of these, 20 participated in a pilot study, while the remaining 301 completed the final questionnaire upon receiving a Google Forms link via WhatsApp. The required sample size for Rasch analysis varies depending on several factors, such as the desired precision of person and item estimates, and the representativeness of the sample relative to the target population. Ideally, an appropriately matched sample should closely mirror the distribution of persons to the distribution of items when calibrated on the same scale. For a well-targeted sample, a sample size of 64 cases is typically sufficient to achieve stable item calibration within ±0.5 logits. For a poorly targeted sample, this number increases to 144 cases to maintain the same level of precision (Ree, 1981). The present study recruited 301 patients with MS.

### Tool validation

Questionnaire validation is an essential step in confirming a research tool's accuracy and consistency. Therefore, the Arabic version of the MSIS-29 underwent a thorough validation process. A panel of experts, consisting of two neurologists, an internist, and a clinical pharmacist, evaluated the content validity of the MSIS-29. The specialists confirmed the questionnaire's thoroughness and comprehensive coverage of the various effects of MS on patients' QoL, all presented in straightforward, simplified language. Twenty MS patients, randomly selected and approached at AL-Bashir Hospital, participated in a pilot study to assess the questionnaire's face validity. They were informed about the purpose of the study, invited to complete the questionnaire, and asked to provide feedback. Patients were invited to participate in an open discussion to evaluate the relevance, clarity, and simplicity of the questionnaire items. The translators and specialists reviewed the feedback and made minor adjustments accordingly, namely, the use of synonym words in three items. The statistical analysis for this study excluded data from the pilot trial. Cronbach's alpha was used to evaluate each scale's internal consistency. Rasch analysis was employed to confirm the construct validity of the tool, evaluate its ability to differentiate between various patients’ QoL levels, and assess the difficulty of the questionnaire's components. Additionally, Wright maps, item fit, person fit, and ability were examined to further assess the tool's quality. Rasch analysis is increasingly being applied due to its ability to assess several properties of latent scales, including a thorough evaluation of the response scale, item content, response bias, dimensionality, and the accuracy of scale targeting (Bond & Fox, 2007; DeVellis & Thorpe, 2022; Pallant & Tennant, 2007). Additionally, it facilitates the assessment of changes in health status by transforming ordinal data into equal-interval measurements (Tennant et al., 2004).

### Statistical analysis

Continuous variables were presented as medians (interquartile range) and categorical variables were presented as frequencies and percentages. A Likelihood Ratio Test was conducted on the MSIS-29-PHYS, MSIS-29-PSYCH, and MSIS-29-TOTAL to determine the most suitable Rasch analysis approach: the Partial Credit Model (PCM) or the Rating Scale Model. Unlike the Rating Scale Model, the PCM does not assume equal distances between answer thresholds and allows items to have different numbers of response categories. If the Likelihood Ratio Test is significant, the PCM is preferred (Ramp et al., 2009) For a scale to fit the Rasch model correctly, it is expected that patients with high levels of the measured characteristic (psychological or physical effects of MS) will consistently select high-scoring response options, while those with low levels will choose low-scoring responses.

Additionally, the response thresholds of every item should be properly ordered. A threshold is the point where two response categories are equally likely to be selected. Disordered thresholds occur when respondents do not consistently choose the correct response alternatives, leading to unreliable responses. This issue may arise from unclear labeling or having too many response options. A threshold map can help identify disordered thresholds, and probability curves for each response type can assess the severity of the problem. To correct disordered thresholds, adjacent response categories can be collapsed if necessary.

The Statistical Package for the Social Sciences (SPSS) version 26, Jamovi version 2.3.28, R (Tam) version 4.0–16, and eRm version 1.0–1 were used for Rasch statistical analysis. To assess the internal validity of the MSIS-29-PHYS, MSIS-29-PSYCH, and MSIS-29-TOTAL, separate Rasch analyses were conducted for each set of items. Each analysis evaluated threshold ordering, overall model fit, item fit, person fit, reliability, differential item functioning (DIF), and targeting. Jamovi 2.3.28 provides the Likelihood Ratio Test to help identify the most appropriate Rasch model for a given dataset. A significant result from this test suggests indicates an inconsistency in the distance between response thresholds, suggesting that the PCM is more suitable.

Model fit was assessed by calculating item and person separation reliability, in addition to Cronbach's alpha, Wright maps, and The Akaike information criterion (AIC). Delta tau parameterization and Thurstone Scale of the PCM were produced to evaluate item and person characteristics and determine the difficulty thresholds for each item. Moreover, DIF was assessed to identify potential item bias related to gender within the sample. A non-significant item-trait interaction chi-square probability value indicated a good overall fit for the model, suggesting that the hierarchical ordering of items remained consistent across all trait levels. Floor/ceiling effects were considered negligible, with effects categorized as significant if they were ≥ 15%, moderate if between 10% and < 15%, minor if between 5% and < 10%, and negligible if < 5%.

## RESULTS

### Demographics

The sociodemographic characteristics of the sample are presented in Table 1. The current study enrolled 301 participants, with a median age of 35 years (range: 28–44), and the majority were women (68.4%). Less than half of the participants (45.2%) held a university degree or higher. Additionally, the majority of patients (68.8%) were unemployed, and 74.4% of participants earned 500 JOD or less per month. Most participants (60.5%) were married, and a vast majority (88.4%) were not studying or working in the medical field. The median disease duration was 6 years (range: 3–10). Finally, 31.9% of the participants had never been hospitalized in the past year, while 13.3% had been hospitalized more than three times.

**Table 1:** Sociodemographic profile of the study participants (N = 301)

		Median (25–75) Or Frequency (%)
Age		35 (28–44)
Gender	Female	206 (68.4%)
	Male	95 (31.6%)
Educational level	High school or less	165 (54.8%)
	University degree	136 (45.2%)
Employment status	Unemployed	207 (68.8%)
	Employed	94 (31.2%)
Income	= < 500 JOD	224 (74.4%)
	> 500 JOD	77 (25.6%)
Marital status	Married	182 (60.5%)
	Not currently married	119 (39.5%)
Working or studying in the medical field	No	266 (88.4%)
	Yes	35 (11.6%)
Disease duration (years)		6 (3–10)
How many times have you been hospitalized last year?	Never	96 (31.9%)
	Once	84 (27.9%)
	Twice	66 (21.9%)
	Three times	15 (5.0%)
	More than three times	40 (13.3%)

### Rasch analysis

Likelihood ratio tests for all three scales were significant, supporting the use of a PCM (MSIS-29-PHYS: *p* < 0.001; MSIS-29-PSYCH: *p* = 0.002; MSIS-29-TOTAL: *p* < 0.001). The Rasch rating scale model showed a poorer fit to the data; hence, the PCM was used for analyzing polytomous responses.

**Fig. 1:**
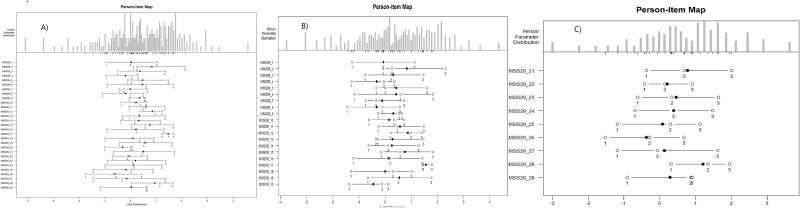
Wright map for (A) MSIS-29-TOTAL, (B) MSIS-29-PHYS, and (C) MSIS-29-PSYCH after reordering.

The Wright map for MSIS-29-PHYS, MSIS-29-PSYCH, and MSIS-29-TOTAL before reordering indicated that all items exhibited some degree of threshold disordering. Several rescoring options were tested, but most disordered thresholds were resolved by reducing the original 5-point response scale (12345) to a 4-point scale (01233), merging the 'extremely' and 'quite a bit' response categories. The rescoring of MSIS-PHYS and MSIS-PSYCH items is shown in Table 2. The only items that retained disordered thresholds based on the Delta-tau parameterization were MSIS-29_13 and MSIS-29_17 (see Table 4). Moreover, the Wright map of MSIS-29-TOTAL illustrates that all items were reordered except for MSIS-29_9, MSIS-29_13, and MSIS-29_17 (see Fig. 1). However, as indicated by the Thurston thresholds, all items were correctly ordered on both scales (see Table 5).

**Table 2:** Reordered frequencies (%) of the Multiple Sclerosis Impact Scale-29 (MSIS-29)

	Not at all	A little	Moderately	Quite a bit
“Physical Impact” Items				
Do physically demanding tasks?	45 (15%)	66 (21.9%)	91 (30.2%)	99 (32.9%)
Grip things tightly (e.g., turning on taps)?	85 (28.2%)	66 (21.9%)	98 (32.6%)	52 (17.3%)
Carry things?	61 (20.3%)	71 (23.6%)	88 (29.2%)	81 (26.9%)
Problems with your balance?	43 (14.3%)	58 (19.3%)	68 (22.6%)	132 (43.8%)
Difficulties moving about indoors?	71 (23.6%)	59 (19.6%)	94 (31.2%)	116 (25.6%)
Being clumsy?	54 (17.9%)	94 (31.2%)	88 (29.2%)	65 (31.2%)
Stiffness?	53 (17.6%)	48 (15.9%)	82 (27.2%)	118 (39.2%)
Heavy arms and/or legs?	40 (13.3%)	61 (20.3%)	76 (25.2%)	124 (41.2%)
Tremor of your arms or legs?	77 (25.6%)	62 (20.6%)	56 (18.6%)	106 (35.2%)
Spasms in your limbs?	64 (21.3%)	64 (21.3%)	65 (21.6%)	108 (35.9%)
Your body not doing what you want it to do?	80 (26.6%)	69 (22.9%)	76 (25.2%)	76 (25.2%)
Having to depend on others to do things for you?	104 (34.6%)	71 (23.6%)	56 (18.6%)	70 (23.2%)
Limitations in your social and leisure activities at home?	67 (22.3%)	48 (15.9%)	100 (33.2%)	86 (28.6%)
Being stuck at home more than you would like to be?	63 (20.9%)	64 (21.3%)	85 (28.2%)	89 (29.6%)
Difficulties using your hands in everyday tasks?	86 (28.6%)	73 (24.3%)	79 (26.2%)	63 (20.9%)
Having to cut down the amount of time you spent on work or other daily activities?	49 (16.3%)	77 (25.6%)	91 (30.2%)	84 (27.9%)
Problems using transport (e.g., car, bus, train, taxi, etc.)?	168 (55.8%)	54 (17.9%)	29 (9.6%)	50 (16.6%)
Taking longer to do things?	47 (15.6%)	77 (25.6%)	78 (25.9%)	99 (32.9%)
Difficulty doing things spontaneously (e.g., going out on the spur of the moment)?	68 (22.6%)	81 (26.9%)	85 (28.2%)	67 (22.3%)
Needing to go to the toilet urgently?	40 (13.3%)	54 (17.9%)	63 (20.9%)	144 (47.8%)
"Psychological Impact" Items				
Feeling unwell?	60 (19.9%)	84 (27.9%)	92 (30.6%)	65 (21.6%)
Problems sleeping?	48 (15.9%)	55 (18.3%)	81 (26.9%)	117 (38.9%)
Feeling mentally fatigued?	48 (15.9%)	72 (23.9%)	98 (32.6%)	83 (27.6%)
Worries related to your MS?	46 (15.3%)	73 (24.3%)	93 (30.9%)	89 (29.5%)
Feeling anxious or tense?	34 (11.3%)	71 (23.6%)	89 (29.6%)	107 (35.6%)
Feeling irritable, impatient, or short-tempered?	24 (8%)	49 (16.3%)	90 (29.9%)	138 (45.9%)
Problems concentrating?	32 (10.6%)	64 (21.3%)	115 (38.2%)	90 (29.9%)
Lack of confidence?	95 (31.6%)	89 (29.6%)	61 (20.3%)	56 (18.6%)
Feeling depressed?	44 (14.6%)	85 (28.2%)	66 (21.9%)	106 (35.2%)

Model fit was assessed by computing item and person separation reliability, Cronbach's alpha (see Table 3), and AIC. Generally, a smaller AIC indicates better predictive performance. For the original data, the AIC for MSIS-29-TOTAL was 21,875, which improved to 18,652 after reordering. For MSIS-29-PHYS, the AIC decreased from 14,617 to 12,480, and for MSIS-29-PSYCH, it improved from 7158 to 6129.

**Table 3:** Item/person separation and Cronbach's alpha for the three scales of the MSIS-29

	Item reliability	Person reliability	Cronbach's alpha
**MSIS-29-PHYS**	0.95	0.92	0.961
**MSIS-29-PSYCH**	0.89	0.84	0.893
**MSIS-29-TOTAL**	0.96	0.95	0.965

**Fig. 2:**
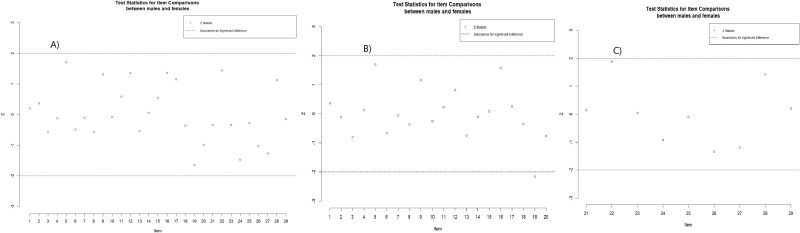
DIF analysis for (A) MSIS-29-TOTAL, (B) MSIS-29-PHYS, and (C) MSIS-29-PSYCH.

Infit and outfit MSQs were within the acceptable range (0.6–1.4) for all items in the MSIS-29-PSYCH scale. However, in the MSIS-29-PHYS scale, item MSIS-29_17 exceeded the acceptable range in both infit (1.93) and outfit (1.82) MSQs, and item MSIS-29_20 exceeded the acceptable range in infit (1.81). The Wright map (Fig. 2) illustrates that item thresholds were roughly evenly distributed, and the participants' status ranged from low to high impact of MS on physical and psychological functioning. Most participants were located in the middle, indicating that the questionnaire effectively differentiated between varying levels of impact. The Wright map and item locations (Table 4) indicated that, on the MSIS-29-PHYS scale, the most common symptom was associated with the item MSIS-29_17, while the least common was associated with the item MSIS-29_20. On the MSIS-29-PSYCH scale, the item MSIS-29_26 represented the most common symptom, and the item MSIS-29_28 represented the least common symptom (Fig. 1). DIF analysis indicated that the locations of the two genders were similar. The z-scores on the MSIS-29-TOTAL showed no differences between genders across all 29 items, demonstrating that all items impacted both genders equally. The same results were observed on the MSIS-29-PSYCH. However, item 19 on the MSIS-29-PHYS slightly exceeded the cut-off z-score (Fig. 2).

**Table 4:** Delta-tau parameterization of the partial credit model for MSIS-29

						tau parameters			
	Outfit mcq	Infit mcq	Measure	1	(SE)	2	(SE)	3	(SE)
Physical Impact Items									
MSIS29_1	1.18	1.10	−0.07	−1.184	(0.22)	−0.0251	(0.19)	1.2096	(0.17)
MSIS29_2	1.23	0.98	0.83	−0.925	(0.18)	−0.5620	(0.18)	1.4872	(0.18)
MSIS29_3	0.89	0.87	0.31	−1.077	(0.20)	−0.0993	(0.16)	1.1768	(0.19)
MSIS29_4	1.34	1.04	−0.32	−0.942	(0.23)	0.2492	(0.19)	0.6927	(0.16)
MSIS29_5	0.71	0.72	0.43	−0.777	(0.20)	−0.4291	(0.18)	1.2057	(0.16)
MSIS29_6	1.06	1.00	0.42	−1.588	(0.20)	0.1579	(0.20)	1.4299	(0.18)
MSIS29_7	0.83	0.94	−0.12	−0.620	(0.23)	−0.2481	(0.19)	0.8676	(0.18)
MSIS29_8	0.74	0.75	−0.32	−1.095	(0.24)	0.1959	(0.18)	0.8994	(0.19)
MSIS29_9	1.35	1.12	0.31	−0.584	(0.19)	0.2262	(0.19)	0.3574	(0.20)
MSIS29_10	1.16	0.94	0.15	−0.775	(0.20)	0.1811	(0.19)	0.5942	(0.17)
MSIS29_11	0.88	0.72	0.57	−0.833	(0.18)	−0.0998	(0.18)	0.9326	(0.17)
MSIS29_12	0.78	0.91	0.87	−0.672	(0.17)	0.1212	(0.18)	0.5504	(0.20)
MSIS29_13	0.88	1.00	0.28	−0.566	(0.21)	−0.6429	(0.17)	1.2093	(0.17)
MSIS29_14	1.00	1.00	0.26	−0.894	(0.20)	−0.1499	(0.24)	1.0436	(0.26)
MSIS29_15	0.64	0.66	0.76	−0.922	(0.18)	−0.1728	(0.17)	1.0944	(0.17)
MSIS29_16	0.93	0.92	0.14	−1.344	(0.21)	0.0447	(0.17)	1.2993	(0.19)
MSIS29_17	1.93	1.82	1.56	−0.156	(0.17)	0.2402	(0.20)	−0.0844	(0.17)
MSIS29_18	0.79	0.78	0.00	−1.295	(0.21)	0.2728	(0.19)	1.0220	(0.17)
MSIS29_19	1.01	1.02	0.54	−1.209	(0.19)	−0.0351	(0.18)	1.2441	(0.18)
MSIS29_20	1.81	1.22	−0.46	−0.892	(0.24)	0.3082	(0.16)	0.5837	(0.19)
Psychological Impact Items									
MSIS29_21	0.63	0.66	−0.0590	−1.177	(0.18)	−0.0701	(0.16)	1.247	(0.20)
MSIS29_22	0.71	0.72	−0.6314	−0.654	(0.22)	−0.0849	(0.19)	0.739	(0.17)
MSIS29_23	0.76	0.79	−0.3788	−1.092	(0.21)	−0.1230	(0.17)	1.215	(0.18)
MSIS29_24	0.82	0.82	−0.4436	−1.108	(0.21)	−0.0159	(0.17)	1.124	(0.18)
MSIS29_25	1.01	0.95	−0.7476	−1.249	(0.24)	0.1761	(0.17)	1.072	(0.17)
MSIS29_26	1.03	0.98	−1.1919	−1.067	(0.30)	0.0057	(0.19)	1.061	(0.16)
MSIS29_27	0.95	1.02	−0.7042	−1.288	(0.25)	−0.2251	(0.17)	1.513	(0.17)
MSIS29_28	1.34	1.13	0.3875	−0.933	(0.16)	0.1910	(0.18)	0.742	(0.23)
MSIS29_29	1.33	1.20	−0.5410	−1.209	(0.21)	0.5615	(0.18)	0.647	(0.18)

**Table 5:** Thurstone thresholds of the partial credit model for MSIS-29-PSYCH

	Thurstone Thresholds		
	1	2	3
Physical Impact Items			
MSIS29_1	−2.081	−0.6741	0.7715
MSIS29_2	−1.088	−0.0539	1.8421
MSIS29_3	−1.631	−0.3339	1.1112
MSIS29_4	−2.103	−0.8112	0.1718
MSIS29_5	−1.357	−0.3696	1.2066
MSIS29_6	−1.905	−0.0761	1.4706
MSIS29_7	−1.755	−0.8182	0.4113
MSIS29_8	−2.231	−0.8233	0.3130
MSIS29_9	−1.210	−0.2077	0.5749
MSIS29_10	−1.517	−0.3766	0.5586
MSIS29_11	−1.184	−0.0671	1.1786
MSIS29_12	−0.722	0.3271	1.2459
MSIS29_13	−1.417	−0.6047	1.0330
MSIS29_14	−1.554	−0.4086	0.9414
MSIS29_15	−1.076	0.0830	1.4825
MSIS29_16	−1.996	−0.4306	1.0612
MSIS29_17	0.339	1.0526	1.6039
MSIS29_18	−2.057	−0.4483	0.7483
MSIS29_19	−1.490	−0.0673	1.4104
MSIS29_20	−2.188	−0.9250	−0.0237
Psychological Impact Items			
MSIS29_21	−1.480	−0.0960	1.398
MSIS29_22	−1.661	−0.6653	0.427
MSIS29_23	−1.742	−0.4419	1.044
MSIS29_24	−1.801	−0.4516	0.921
MSIS29_25	−2.190	−0.6569	0.610
MSIS29_26	−2.513	−1.1892	0.127
MSIS29_27	−2.241	−0.8334	0.957
MSIS29_28	−0.800	0.4705	1.501
MSIS29_29	−1.903	−0.2870	0.589

## DISCUSSION

The current study aimed to assess the validity of the MSIS-29 Arabic version for MS patients in Jordan. The findings show that the Arabic version of MSIS-29 effectively discriminates between different levels of quality of life among participants. The data proved suitable for Rasch analysis, confirming that the translated MSIS-29 accurately captured the impact of MS on Jordanian participants. By reordering the scale, we were able to enhance its precision, thereby increasing the reliability of the tool for assessing the nuances of disease impact. The model fit, Cronbach's alpha, Wright map, and AIC collectively demonstrate that the Arabic version of the tool effectively discerned different levels of quality of life among participants. The psychometric evaluation of the MSIS-29's individual scales, MSIS-29-PHYS and MSIS-29-PSYCH, shows robust support. With a good fit to the Rasch model, absence of differential item bias, high internal consistency, and appropriate targeting of the scales, the study confirms the reliability and validity of each scale. These findings underline the scales' effectiveness in accurately measuring the specific physical and psychological impacts of MS on patients.

In a comprehensive Rasch analysis of the MSIS-29, Bacci et al. reported that disordered thresholds in both the psychological and physical impact subscales rendered the five-category scoring system unsuitable for their community-based population (Bacci et al., 2016). Similarly, Ramp et al. found that 11 of the 20 physical impact items exhibited some threshold disordering following their Rasch evaluation, suggesting a need for revision in the response options (Ramp et al., 2009). These findings are consistent with the present study, where the original response scales showed disordering across all items. However, after reordering, all items on both scales were correctly ordered, apart from the items MSIS-29_13 and MSIS-29_17 in the MSIS-29-PHYS scale. This consistency across studies underlines the necessity for continuous refinement of the MSIS-29 to ensure its accuracy and relevance in varied settings.

The infit and outfit indices from this study indicate that the MSIS-29-PHYS and MSIS-29-PSYCH scales generally fit well, with all items within the acceptable range except for the item MSIS-29_17 on the MSIS-29-PHYS. Contrastingly, Ramp et al. noted misfits for the items MSIS-29_20 on MSIS-29-PHYS and MSIS-29_22 on MSIS-29-PSYCH (Ramp et al., 2009). Similarly, Bacci et al. observed substantial misfits, with 33% of the MSIS-29-PSYCH items and 30% of the MSIS-29-PHYS items failing to fit well, particularly the items MSIS-29_18 and MSIS-29_20 (Bacci et al., 2016). These discrepancies between studies suggest that the suitability of the MSIS-29 may vary across different populations and emphasize the importance of continual testing and adaptation of the scale to ensure its effectiveness and relevance.

In this study, the item distribution for the MSIS-29-PHYS showed that the most reported impact, based on the lowest item location, was associated with MSIS-29_20 (“Needing to go to the toilet urgently?”). Conversely, the least common impact, indicated by the highest item location, was MSIS-29_17 (“Problems using transport (e.g., car, bus, train, taxi, etc.)?”). For the MSIS-29-PSYCH, the most frequently reported issue was MSIS-29_26 (“Feeling irritable, impatient, or short-tempered?”), while the least frequent was MSIS-29_28 (“Lack of confidence?”).

Comparatively, Ramp et al. (Ramp et al., 2009) found different patterns in their study. On the MSIS-29-PHYS, the most common issue was MSIS-29_1 (“Do physically demanding tasks?”), and the least common was MSIS-29_5 (“Difficulties moving about indoors?”). On the MSIS-29-PSYCH, the most frequently reported concern was MSIS-29_24 (“Worries related to your MS?”), with the same item, MSIS-29_28 (“Lack of confidence?”), being the least common, as in the current study.

The present study confirmed that the two scales of the MSIS-29 measure correlated yet distinct constructs, as evidenced by the high inter-correlation (rho = 0.769) between the physical and psychological scales. This finding suggests a circular relationship between psychological and physical impacts and aligns with research which reported a correlation of rho = 0.62 (Ramp et al., 2009). The higher correlation between the two scales detected in the present study could be attributed to the nature of Arabic culture where psychological manifestations are taboo and might instead be reported as, or even experienced as, physical symptoms to avoid stigma (Al-Krenawi, 2005). Nevertheless, these results support the argument against using a combined total scale score for epidemiological and clinical research, given the distinct nature of the constructs measured (Hobart et al., 2001).

Floor/ceiling effects, defined as the percentage of respondents who receive the highest (ceiling) or lowest (floor) possible scores in any given domain, were found to be negligible. This demonstrates the questionnaire's capacity to discern between respondents at the extremes of the scale, indicating robust sensitivity and coverage at both ends. For instance, a high floor percentage might indicate that many patients scoring at the lowest possible level on the questionnaire share similarly severe health conditions, suggesting that the instrument struggles to distinguish among patients at the lower end of the scale. Similarly, pronounced floor/ceiling effects could also signal potential issues like response bias, measurement errors, or a restricted instrument range, all of which point to inadequate questionnaire performance (Gulledge et al., 2019).

Tools such as the MSIS-29 are vital for understanding the complex effects of diseases like MS. Given MS’s varied symptoms, its unpredictable progression, its widespread occurrence, and the absence of a cure, detailed assessments are especially vital. This study reaffirms the validity of the MSIS-29's scales for both physical and psychological impacts, highlighting their importance in both clinical and research settings to enhance our understanding of the disease’s effects. Additionally, our results emphasize the value of using Rasch analysis to ensure these scales accurately assess what they are meant to measure, which is essential for reliable patient evaluation.

### Strengths, limitations, and future directions

The current study benefited from a robust sample size of 301 patients and employed the well-regarded Rasch analysis methodology, ensuring reliable and valid findings regarding the performance of the MSIS-29 across its physical and psychological scales. However, a limitation of this study is that all participants were recruited from a single hospital. Although this hospital serves a large geographic area in Jordan, the findings may not be generalizable to all MS patients, particularly those in different regions or healthcare settings. Another limitation that may affect the generalizability of the study’s results is that nearly half of the sample had a university degree, which may limit the applicability of the findings to less educated populations. Additionally, the reliance on self-reported data introduced the potential for recall bias, as participants may not have accurately remembered or reported their symptoms and experiences.

Future research should aim to include a more diverse patient population from multiple hospitals or regions to enhance the generalizability of the results. It would also be beneficial to incorporate longitudinal data to observe changes over time and reduce the impact of recall bias. Furthermore, integrating objective measures of disease progression and symptom severity could complement the self-reported data, providing a more rounded assessment of the MSIS-29’s effectiveness. Another promising area for research could be the application of the MSIS-29 in cross-cultural settings, which would help refine the tool for global use and ensure its sensitivity across different cultural contexts.

## CONCLUSION

The Arabic version of the MSIS-29 effectively captured both the physical and psychological effects of MS from the patients' viewpoints. Our findings confirm that this tool is both valid and reliable for assessing the impact of the disease and the quality of life among MS patients in Jordan. Moreover, these results endorse the use of the MSIS-29 as an outcome measure in a variety of clinical settings, which can help improve healthcare services. This reinforces the significance of the MSIS-29 in understanding patient experiences and enhancing patient care across diverse medical environments.
