# Correction to: Validation of the Arabic Version of the Multiple Sclerosis Impact Scale (MSIS-29): a Rasch Analysis Study

**DOI:** 10.1093/arclin/acaf059

**Published:** 2025-06-06

This is a correction to: Walid Al-Qerem, Dunia Basem, Sawsan Khdair, Anan Jarab, Judith Eberhardt, Validation of the Arabic Version of the Multiple Sclerosis Impact Scale (MSIS-29): a Rasch Analysis Study, *Archives of Clinical Neuropsychology*, Volume 40, Issue 3, May 2025, Pages 520–528, https://doi.org/10.1093/arclin/acae121

In the originally published version of this paper, the author Anan Jarab was incorrectly listed as being affiliated with the Department of Clinical Pharmacy, Faculty of Pharmacy, Jordan University of Science and Technology. This affiliation has been removed.

